# MCCL: an open-source software application for Monte Carlo simulations of radiative transport

**DOI:** 10.1117/1.JBO.27.8.083005

**Published:** 2022-04-12

**Authors:** Carole K. Hayakawa, Lisa Malenfant, Janaka Ranasinghesagara, David J. Cuccia, Jerome Spanier, Vasan Venugopalan

**Affiliations:** a University of California at Irvine, Department of Chemical and Biomolecular Engineering, Irvine, California, United States; b University of California at Irvine, Beckman Laser Institute, Irvine, California, United States; c Modulim, Irvine, California, United States

**Keywords:** Monte Carlo simulation, open-source software, photon migration, diffuse optics

## Abstract

The Monte Carlo Command Line application (MCCL) is an open-source software package that provides Monte Carlo simulations of radiative transport through heterogeneous turbid media. MCCL is available on GitHub through our virtualphotonics.org website, is actively supported, and carries extensive documentation. Here, we describe the main technical capabilities, the overall software architecture, and the operational details of MCCL.

## 1 Introduction

Monte Carlo (MC) methods to provide solutions for the radiative transport equation (RTE) originated at Los Alamos National Lab in 1946. At that time, the intent was to solve nuclear fission problems. The initial code base that was developed has subsequently evolved into the Monte Carlo N-Particle Transport (MCNP^1^) code.^2^ Since then, MC simulations of radiative transport have been used to solve problems in biomedical optics. The first paper documenting the use of MC to study light propagation in tissue was published by Wilson and Adam,^3^ and this was followed by Prahl et al.^4^ which served as a precursor to the seminal 1995 paper by Wang et al.^5^ that introduced the open-source MC Command Line application (MCML) code commemorated in this special issue.

Our development of the MCCL began with C code developed initially by Andy Dunn and Derek Smithies at the Beckman Laser Institute (BLI) at the University of California, Irvine, in 1998. Carole Hayakawa, under the direction of Jerry Spanier, advanced this code base through the development of perturbation and differential MC methods in support of her doctoral thesis.^6^^,^^7^ In 2008, the Virtual Photonics Technology Core (VPTC) was formed within the BLI National Institutes of Health (NIH) P41 Biomedical Technology Research Center (Laser Institute Laser Microbeam and Medical Program) under the leadership of Jerry Spanier and Vasan Venugopalan. The formation of the VPTC motivated the systematic organization of the software with the intent of open-source distribution to the Biomedical Optics community. This effort coincided with a NIH K25 award to Hayakawa to further develop a “Virtual Tissue Simulator for Biomedical Optics.” Soon after David Cuccia saw multiple opportunities to restructure the code to enable: (a) an object-oriented and extensible software platform and (b) organized versioning that would provide a stable code base for students/researchers who may require specialized code modifications for their work. Cuccia and Hayakawa ported the code to C# and the first version of the MCCL was uploaded to CodePlex in 2010. We migrated the code to GitHub (https://github.com/VirtualPhotonics/Vts.MonteCarlo/wiki) in 2017 where it has been maintained ever since. In 2014, Ranasinghesagara added a diverse set of optical source definitions suited for a variety of applications (see Secs. 2.7 and 3.3 for more information). A unique aspect of our open-source code project is our commitment to have professional management of our code and web development, a critical role that Lisa Malenfant has provided since 2010. This ensures that our code has guidelines, naming conventions, documentation, and a web presence that is informative and easy to navigate. From 2013 to 2018, the VPTC led the development and delivery of annual Short Courses in Computational Biophotonics (https://education.virtualphotonics.org). In these courses, MCCL played an integral role in the lab exercises to demonstrate the capabilities and characteristics of MC simulations to solve the RTE and illustrate important conceptual aspects of light transport within biological tissues.

We developed the MCCL to provide the following capabilities: (a) software extensibility to allow easy incorporation of additional source, tissue, and detector options, (b) various optical sources and detectors as well as commonly analyzed tissue structures used in the biomedical optics community, and (c) simulations using foundational MC methods, e.g., those employed by MCNP, and advanced methods, such as perturbation MC. Our objective in this paper is to describe our open-source MC simulation engine, its modular architecture, features, operation, and the ample resources we have provided to support user installation and usage.

## 2 Important Features of MCCL

MCCL is packaged with the Monte Carlo Post Processor (MCPP) and is available for Windows, MacOS, and Linux by downloading the software from the Virtual Photonics GitHub website (https://github.com/VirtualPhotonics). Directions for downloading a zip file with executables and for cloning and building it from source code are provided on the “wiki” pages of the MCCL GitHub website (https://github.com/VirtualPhotonics/Vts.MonteCarlo/wiki). For downloading a zip file, there are separate instructions for Linux (https://github.com/VirtualPhotonics/Vts.MonteCarlo/wiki/MCCL-Getting-Started-on-Linux), Mac (https://github.com/VirtualPhotonics/Vts.MonteCarlo/wiki/MCCL-Getting-Started-on-Mac), and Windows (https://github.com/VirtualPhotonics/Vts.MonteCarlo/wiki/MCCL-Getting-Started-on-Windows). All operating systems require .NET 5.0 consistent with the respective system.

The code is written in C#, an object-oriented, strongly typed language that allows the software to be easily extensible. Moreover, C# is a component-oriented language which means that the code can be written in the form of components that can be interchanged. In particular, our MCCL design permits the use of any combination of sources, tissues, detectors, and MC estimators within a single simulation. Both MCCL and MCPP have been adapted for .NET Core, and therefore cross-platform applications, which allows a single source to generate executables that run on Windows, Mac, and Linux. In addition, MCCL can be executed across multiple CPUs for improved computational efficiency.

### 2.1 Open-Source

MCCL and MCPP are both open-source and maintained in a software repository that provides version control for releases including detailed release notes. The software generated by the Virtual Photonics Technology Initiative (VPTI; https://virtualphotonics.org) is hosted on GitHub (https://github.com/VirtualPhotonics), a website that hosts open-source software. On GitHub, we provide wiki pages that document the software, an issues section where users can communicate with the development team regarding issues with the software, and instructions on how to pull the source code and download the latest executables. Subpages provide information regarding how to edit input files, examples of command line directives, capabilities, and implementation, detail on source definitions, instruction on operating the post-processor and executing inverse solutions, and a frequently asked questions page. We also list all references used in the code.

### 2.2 Software Interfaces

A key advantage of the MCCL and MCPP software architecture is C#’s use of classes and interfaces to readily provide extensibility of the software. A software class is a user-defined blueprint or prototype from which objects are created and an interface looks, such as a class, but has no implementation. Interfaces can contain declarations of fields, properties, and methods. These declarations specify a software contract between the interface and any class that implements it. The contract specifies that the class will implement the fields, properties, and methods and their signatures defined in the interface. Once an interface is defined, coding a specific class that implements the interface is clearly defined to readily provide software extensibility. We have designed all our source, tissue, and detector classes with interfaces to simplify future additions. Our most commonly used sources and detectors are listed in tabular form in Sec. 3.3. Moreover, the use of interfaces provides for easier code maintenance since the interface contract prevents any future implementations of classes from breaking the code. Some of the interfaces defined for MCCL and MCPP include: the absorption weighting methods, inputs for optical sources, detectors and tissues, and the resulting class implementations. For example, our TissueInput interface specifies a TissueType identifier string, an array of tissue Regions, and a method to create the corresponding tissue, CreateTissue. Any new tissue class added to the software would implement this interface, and therefore, be required to have code that defines the TissueType, Regions, and a method CreateTissue. The use of interfaces allows users to pipeline together a selected source, tissue, and detectors into a simulation requiring little knowledge of code.

### 2.3 Multiprocessor Capability

MCCL provides the option to execute simulations in parallel across a specified number of CPUs. This can be invoked using the option “cpucount=#” in the command line. Usage of the multiprocessor capability requires the selection of a random number seed in each simulation such that the random number sequences on each CPU or thread are independent, i.e., do not overlap. When executing the code on a single CPU, we employ the Mersenne Twister algorithm^8^ which generates pseudo-random numbers with period $2^{19937}$. When executing on multiple CPUs, the code uses a Dynamic Creator Mersenne Twister^9^ which finds substreams of the original Mersenne Twister to ensure that the substreams are independent.

### 2.4 Tissue Heterogeneities

MCCL supports MC simulations in layered tissues to model tissue structures such as skin as well as the introduction of inclusions, such as ellipsoids, cubes, and cylinders, with different optical properties to approximate internal structures such as tumors or blood vessels. Our use of analytical functions to define inclusions was inspired by the Los Alamos National Lab MCNP code. The usage of analytic functions to define the boundaries of the heterogeneities provides two key advantages. First, the means to determine whether the photon is inside or outside the heterogeneity can be performed through the evaluation of a quadratic function. Second, the curved surfaces of heterogeneities, such as spheres, ellipsoids, or cylinders are modeled exactly and avoids the “voxelization” errors that can occur when modeling objects with curved surfaces that lead to significant errors in computation of reflectance and transmission across interfaces.^10^ A notable drawback of this approach is that heterogeneous tissues defined by a network of tetrahedrons cannot be modeled,^11^^–^^13^ and Digital Imaging and Communications in Medicine images cannot be currently imported to define the tissue geometry. However, a key objective in developing MCCL was to develop a modular MC simulation platform that rigorously solves the RTE to support streamlined investigations for a wide variety of simple model problems and can examine the impact of different optical sources, detectors, and/or MC estimators on performance and computational efficiency. MCCL is not intended to serve as a medical imaging tool as there are other code bases that fill that need.

### 2.5 Variance and Efficiency

Because MC is a stochastic solution method, the results of all simulations have an associated uncertainty or variance that quantifies their accuracy. In our design of MCCL, we have prioritized direct user access to rigorous metrics for the uncertainty of the simulation output so that they can properly interpret MCCL results. The fundamental metric characterizing the uncertainty of a MC random variable ξ is the variance defined as $\mathrm{Var}(\xi )=\frac{1}{N}\underset{N}{\sum }{\left(\xi -E[\xi ]\right)}^{2}$, where E[ξ] is the expected value or mean of ξ. This is equivalent to the second central moment of ξ. The standard deviation of the random variable σ(ξ) is given by the square root of the variance. Users can generate the standard deviation associated with the tally from each detector by setting “TallySecondMoment” to “true” in the input file. A rigorous comparative analysis of the performance of two different MC simulations is provided by a metric called computational efficiency. The computational efficiency Eff[ξ] of ξ is given by $1/[R^{2}T]$ where R is the relative error and T is the computer run time.^2^ The relative error R[ξ] in the estimate of ξ is given by $\frac{\sigma }{E[\xi ]}$.

### 2.6 Absorption Weighting Types

All MC codes require a method to account for optical absorption. MCCL allows the user to specify the use of Analog, Discrete Absorption Weighting (DAW), or Continuous Absorption Weighting (CAW).^14^ The desired weighting method can be selected by setting “AbsorptionWeightingType” to “Analog,” “Discrete,” or “Continuous.” Each method has advantages and disadvantages depending on the system under investigation.^15^ In the Analog method, the photon retains a weight of 1, however, at each scattering event it can be absorbed and terminated with probability $\left(\mu_{a}/\mu_{t}\right)$. Intercollision distances are sampled from the distribution $\mu_{t}\;\exp(-\mu_{t}s)$ where s is the track length between collisions. Using this method can lead to infrequent tallies at detectors located distally from the source which result in MC estimates with high variance.^2^ DAW and CAW are absorption weighting techniques that reduce the weight of the photon during propagation to account for absorption and disallow termination of the photon until it exits the system. DAW which is widely used in other MC programs including MCML, bases intercollision distances on the distribution $\mu_{t}\;\exp(-\mu_{t}s)$, where s is the track length, while CAW bases these distances on $\mu_{s}\;\exp(-\mu_{s}s)$.^15^ All of these absorption estimators are unbiased so they provide random variables that converge to the solution of the RTE in the limit as N (the number of photons launched) goes to infinity.^14^

Most MC codes in the biomedical community enable the use of DAW and/or CAW, however, none to our knowledge enable Analog^14^ processing. In terms of computational efficiency, estimation of ξ using an Analog random walk process can provide higher efficiencies compared to DAW and CAW due to the simplicity of the computations involved which result in shorter computer run times. However, for a given problem it is difficult to know in advance which estimator will produce the greatest efficiency.^15^

### 2.7 Sources

MCCL provides numerous input options that enable the simulation of both external optical sources irradiating the tissue surface or interstitial sources that reside within the tissue volume. The input options provide the flexibility needed to accurately model physical optical sources. MCCL allows the user to define basic sources, such point and line sources, but also surface and volumetric sources, such as surface emitting sources (emitted from flat circular, elliptical, or rectangular surfaces), surface emitting bulk sources (emitted from cylindrical fiber, spherical, cuboidal, or tubular surfaces), and volumetric sources (emitted from the volume of a cube, or tissue region for fluorescence emission). Our extensive selection of optical sources provides the ability to tailor spatial and angular characteristics and is described further in Sec. 3.3.

### 2.8 Detectors

MCCL provides options for detectors that tally upon the exit of photons from the tissue, i.e., reflectance and transmittance, and detectors that require data associated with the photon trajectory to determine a tally internal to the tissue, i.e., absorbed energy, fluence, radiance, and momentum transfer.^16^ The detector spatial bins can be defined in terms of Cartesian and/or cylindrical grid specifications. Angular bins can be custom designed using polar and azimuthal bin specifications. MCCL provides detectors supporting common spatial and/or temporal detection schemes, i.e., spatially resolved (based on source–detector separation), spatial-frequency domain (for spatially modulated sources), temporally resolved, or temporal-frequency domain (for intensity-modulated sources).

The MC estimators for the spatial-frequency and temporal-frequency domains are generated by forming a complex photon weight from analysis of spatial and temporal Fourier transforms of the RTE. For both domains, modified shortcut methods^17^^,^^18^ are used to provide results for multiple spatial or temporal frequencies derived from a single MC simulation.

### 2.9 Databases and Photon Biography Postprocessing

MCCL enables the user to save photon trajectory information into a database that can be post-processed later by the MCPP. For example, the user can specify in the input file for a “DiffuseReflectance” database to be generated. This database stores the photon exiting position, direction, exiting weight, and time of flight prior to exit for each of the photons exiting the tissue surface.

Such a database can serve many uses. The user can examine the impact of bin size (angular, spatial, temporal, and spatial/temporal frequency) on the resulting tally and its variance. Moreover, such a database can be postprocessed to obtain other tallies at a fraction of the computational cost compared to an independent MC simulation.

### 2.10 Perturbation and Differential Monte Carlo

Perturbation Monte Carlo (pMC) and differential Monte Carlo (dMC) estimates can also be provided by postprocessing a database that compiles the collision locations and length of track segments for each of the detected photons.^19^ Perturbation MC enables the user to obtain rigorous estimates of the change in response of any detector for a specified (small) change in the tissue optical properties without the need to run separate MC simulations. This is performed by using the random walks of a simulation based on the baseline tissue optical properties and appropriately re-weighting the random variable using the Radon–Nikodym derivative.^20^ The Radon–Nikodym derivative also enables the determination of derivatives^7^ of a measured signal relative to changes in the optical properties. This capability provides an efficient means for sensitivity analysis and performing a gradient-based optimization for the resolution of inverse problems.^6^^,^^7^^,^^21^^–^^26^ The ability to compute pMC and dMC estimates within a MC simulation and use them for sensitivity analyses or inverse solutions represents a unique aspect of our software. Further detail and depiction of our pMC/dMC software design is provided in Sec. 3.5 with sample results provided in Sec. 4.

## 3 Architecture and Usage of MCCL

### 3.1 Software Architecture

**Fig. 1:**
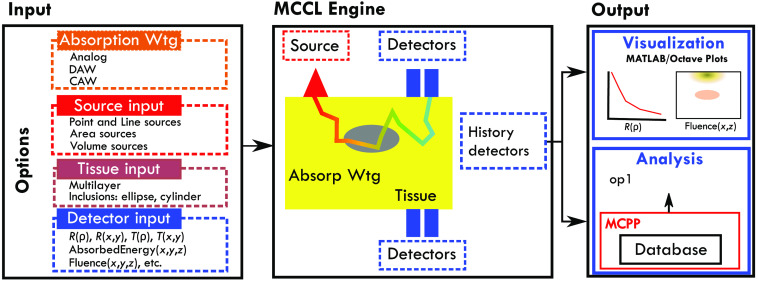
Software architecture: dashed lines represent interfaces and example classes that implement the interfaces (see Sec. 2.2). (a) Input: user specifications of system under investigation, (b) MCCL Engine: random walk process designated by input including photon weight decay during random walk process via absorption weighting specification, and detector tallies, and (c) Output: visualization of detector results and/or analysis of results.

The MCCL framework provides the user with the ability to (a) specify inputs necessary to define a simulation (Input), (b) execute the simulation (MCCL Engine), and (c) visualize (MATLAB^27^/Octave^28^ plots), and/or further analyze (MCPP) simulation results. Specific software interfaces have been designed to provide each of these functionalities. A subset of the functionality provided by these three components is schematically shown as rectangles with dashed lines in Fig. 1. Sample classes that implement these interfaces are shown within some rectangles.

The MCCL input file is written in JavaScript Object Notation (JSON) and is read by the MCCL Engine. The input is comprised of three major sections that allow the user to specify the simulation source, tissue, and detectors, each which have interface definitions. Sample input files are shown on our GitHub website Sample Input & Output (https://github.com/VirtualPhotonics/Vts.MonteCarlo/wiki/MCCL-Sample-Input-And-Output). At the start of the file there is also an “OutputName” that designates the folder where the results will be written, “N” the number of photons to launch and “Options” that specify various options to the simulation. The detectors can be specified on the tissue surface, reflectance, transmittance, emitted radiance, or history detectors that capture internal tallies such as, absorbed energy, radiance, or fluence. The internal tallies are referred to as “history detectors” because they require information from all interactions that each photon experiences along its trajectory.

The detector output is written to binary files that can be plotted and visualized using MATLAB or Octave and/or saved to a database for further analysis using MCPP (see Sec. 3.5). Analysis results include perturbation MC results, e.g., estimation of the perturbed reflectance due to $\mu_{a}$ and/or $\mu_{s}$ changes in any/all regions of the tissue $R^{*}(\mu_{a}+\Delta \mu_{a},\mu_{s}+\Delta \mu_{s})$, and differential MC estimates of the derivative of reflectance with respect to $\mu_{a}$ and/or $\mu_{s}$, $\partial R^{*}/\partial \mu_{a}$, and $\partial R^{*}/\partial \mu_{s}$. The pMC and dMC detector results can be generated by specifying them in an MCCL input file or by generating a database and specifying them in an MCPP input file. MATLAB/Octave script files to perform the plotting are provided with the software.

### 3.2 MCCL Inputs: Options

The “Options” section of the input file contains various settings that specify the desired characteristics of executing the transport of photons through the tissue. All settings are described in this section in the order they appear in the input file.

Setting the “Seed” to 0 or a greater integer will generate a reproducible stream of random numbers. Setting the seed to −1 will designate a random stream of random numbers each time the simulation is executed.

The “RandomNumberGeneratorType” requires the user to specify the “MersenneTwister.”^8^

Setting “AbsorptionWeightingType” to “Analog,” “Discrete,” or “Continuous” will specify application of Analog, DAW, or CAW random walk processes to account for absorption, respectively.

The “PhaseFunctionType” specifies the probability distribution function governing the photon deflection angle for scattering. Currently implemented options are “HenyeyGreenstein” which is typically used in tissue optics problems^29^ or “Bidirectional,” which can be used to model one-dimensional (1D) slab problems.

The “Databases” options provide the ability to save information regarding photon trajectories to enable subsequent analysis/post-processing of the simulated photons. Setting this option to “DiffuseReflectance” will generate a binary database that includes pertinent photon data such as the exiting location, direction, and weight of the photon. This database can be post-processed in a few seconds to produce any of our reflectance detector estimates (Sec. 2.8).

pMC and dMC estimates are invoked by setting “Databases” to “pMCDiffuseReflectance.” This setting is possible only when the “AbsorptionWeightingType” is set to “Discrete” or “Continuous.” In addition to the photon exiting data when using the “DiffuseReflectance” option, a “CollisionInfo” database is generated that saves the photon path length and number of collisions in each tissue region. When the “AbsorptionWeightingType” is set to “Continuous” and only $\mu_{a}$ changes within any tissue region are specified, Beer’s law is then applied to determine the final weight. pMC is utilized in all other cases, i.e., for absorption or scattering perturbations using DAW or scattering perturbation using CAW. pMC and dMC can be used together to solve inverse problems through the use of a gradient-based optimization algorithm.^6^^,^^7^^,^^21^^–^^23^^,^^25^^,^^26^ Further details are provided in Sec. 4.1.

Setting “TrackStatistics” to true designates that a “statistics.txt” file will be created at the end of the simulation. This file will list the number of photons that (a) exit the top surface of the tissue, (b) exit the bottom surface of the tissue, (c) are specularly reflected by the tissue surface and do not propagate within the tissue volume. Moreover, if Russian Roulette (RR) is employed by seeing the option “RussianRouletteWeightThreshold” to a positive, non-zero value, this file will list the number of photons killed by RR. Such statistics can be valuable to diagnose potential problems in the setup of a simulation and/or understand unexpected results. Moreover, conservation of photon weight (energy) can be verified through examination of these numbers.

The “RussianRouletteWeightThreshold” specifies the threshold value of the photon weight that invokes RR. Russian Roulette is an unbiased method for reducing the run time of a simulation by killing off photons once their weight falls below a specified threshold with a fair game probability.^2^ The rule governing the fair game probability once the photon weight falls below the threshold is to amplify the photon weight by the factor (1/P) with probability P, or kill the photon with probability 1−P. The usage of RR is only possible for MC simulations using either discrete or continuous absorption weighting (Sec. 2.6).

The “SimulationIndex” identifies the output from the simulation in the case where multiple simulations are running at the same time, e.g., when performing a parameter sweep or when multiple processors are specified (Sec. 3.4).

### 3.3 MCCL Inputs: SourceInput, TissueInput, and DetectorInput

We offer an extensive list of source, tissue, and detector inputs to MCCL users. The full list of source, tissue, and detector inputs can be found in an online document, “Source, Tissue, Detector Parameters,” in the right-hand menu of our MCCL GitHub wiki (https://github.com/VirtualPhotonics/Vts.MonteCarlo/wiki). In this section, we describe a subset of the options. Table 1 gives a partial list of the source options available in MCCL.

**Table 1:** Partial list of MCCL source specifications identified by their name and options. The columns refer to the independent variables provided by these sources in terms of source geometry specific definitions, location with optional translation from origin (0,0,0), direction with optional rotation about principal axis, beam rotation about principal axis, polar (θ) or azimuthal (ϕ) emission, angle of convergence or divergence (θ conv or div), source profile (Gaussian or flat) and in what region is the source initiated (initial tissue region). Details can be found on our MCCL GitHub wiki.

Source name	Geometry	Location: any	Location: translate	Direction: any	Direction: rotate	Beam rotation	θ emission	ϕ emission	θ conv or div	Source profile	Initial tissue region
DirectionalPoint	Point	✓	—	✓	—	—	—	—	—	—	✓
DirectionalLine	Length	—	✓	—	✓	✓	—	—	—	✓	✓
DirectionalCircular	Ring radii	—	✓	—	✓	✓	—	—	✓	✓	✓
DirectionalElliptical	a and b axes	—	✓	—	✓	✓	—	—	✓	✓	✓
DirectionalRectangular	Length and width	—	✓	—	✓	✓	—	—	✓	✓	✓
CustomPoint	Point	✓	—	✓	—	—	—	✓	—	—	✓
CustomLine	Length	—	✓	—	✓	✓	—	✓	—	✓	✓
CustomCircular	Ring radii	—	✓	—	✓	✓	—	—	—	✓	✓
CustomElliptical	a and b axes	—	✓	—	✓	✓	—	—	—	✓	✓
CustomRectangular	Length and width	—	✓	—	✓	✓	—	—	✓	✓	✓
CustomVolumetricEllipsoidal	a, b, and c axes	—	✓	—	✓	✓	✓	✓	—	✓	✓
CustomVolumetricCuboidal	Length, width, and height	—	✓	—	✓	✓	✓	✓	—	✓	✓
IsotropicPoint	Point	✓	—	✓	—	—	—	—	—	—	✓
IsotropicLine	Length	—	✓	—	—	—	—	—	—	✓	✓
IsotropicVolumetricEllipsoidal	a, b, and c axes	—	✓	—	✓	✓	—	—	—	✓	✓
IsotropicVolumetricCuboidal	Length, width, and height	—	✓	—	✓	✓	—	—	—	✓	✓
LambertianSurfaceEmittingSpherical	Radius	—	✓	—	—	—	—	—	—	—	✓
LambertianSurfaceEmittingCubiodal	Length, width, and height	—	✓	—	✓	✓	—	—	—	✓	✓
LambertianSurfaceEmittingTubular	Radius and height	—	✓	—	✓	✓	—	—	—	✓	✓
FluorescenceEmissionAOfXAndYAndZ	A(x,y,z)	—	—	—	—	—	—	—	—	—	—

**Fig. 2:**
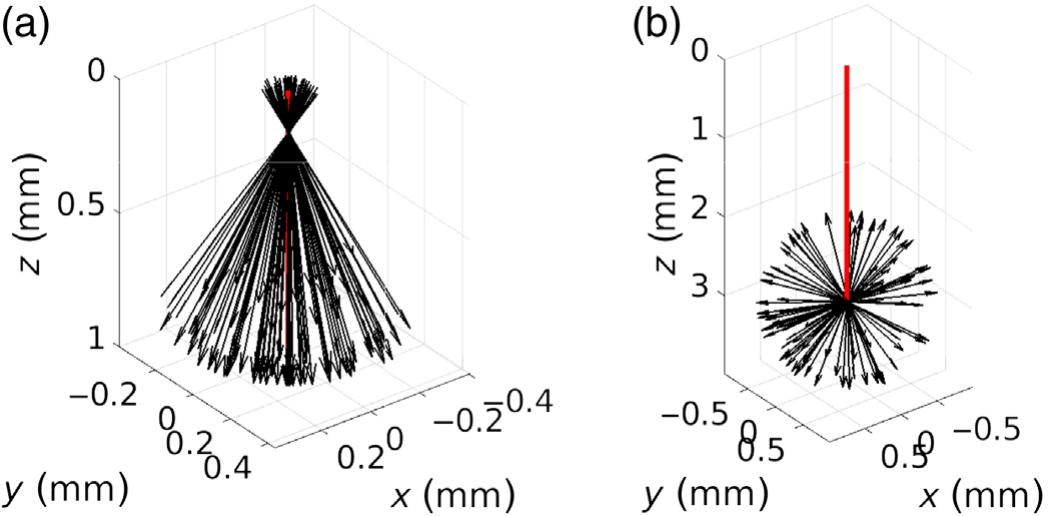
Example source implementations showing 100 initial unit directional vectors emanating from the source for (a) a surface directional circular source (DirectionalCircular) with radius 0.1 mm, converging with angle of 30 deg, and (b) an internal isotropic point source (IsotropicPoint) at depth z=3  mm. The principal axis is shown in red and the z=0 plane is the tissue surface.

Details of our various source configurations (e.g., source code, graphical representations, and equations that are used to sample the source) are described in an online document, “Source Definitions,” in the right-hand menu of our MCCL GitHub wiki (https://github.com/VirtualPhotonics/Vts.MonteCarlo/wiki). Figure 2 shows two source models available in MCCL, the external or internal location of the point source and the initial tissue region index.

MCCL provides detectors to tally photon propagation as a function of space, time, spatial frequency, and temporal frequency with user control over the range and resolution of the independent variable of interest. Tables 2 and 3 provide partial lists of the reflectance and history detector options, respectively, available in MCCL. A full list for reflectance, transmittance, and history detectors can be found in an online document, “Source, Tissue, Detector Parameters,” in the right-hand menu of our MCCL GitHub wiki (https://github.com/VirtualPhotonics/Vts.MonteCarlo/wiki).

**Table 2:** Partial list of MCCL reflectance detector specifications identified by their name and options. The columns refer to the independent variables provided by these detectors in space (ρ,x,y,z), maximum depth ($z_{\max}$), spatial-frequency ($f_{x}$), time (t), temporal-frequency (Ω), and/or polar, and azimuthal angles (θ,ϕ).

Reflectance detector name	ρ∈[0,∞) [mm]	$z_{\max}\in [0,\infty )$ [mm]	x,y∈(−∞,∞) [mm]	$f_{x}\in [0,\infty )$ $\left[{\mathrm{mm}}^{-1}\right]$	t∈[0,∞) [ns]	Ω∈[0,∞) [GHz]	θ∈[π/2,π] [rad]	ϕ∈[0,2π] [rad]
RDiffuse	—	—	—	—	—	—	—	—
ROfAngle	—	—	—	—	—	—	✓	—
ROfRho	✓	—	—	—	—	—	—	—
ROfRhoAndAngle	✓	—	—	—	—	—	✓	—
ROfRhoAndTime	✓	—	—	—	✓	—	—	—
ROfRhoAndOmega	✓	—	—	—	—	✓	—	—
ROfRhoAndMaxDepth	✓	✓	—	—	—	—	—	—
ROfXAndY	✓	—	✓	—	—	—	—	—
ROfXAndYAndMaxDepth	✓	✓	✓	—	—	—	—	—
ROfXAndYAndTime	✓	—	✓	—	✓	—	—	—
ROfXAndYAndThetaAndPhi	—	—	✓	—	—	—	✓	✓
ROfFx	—	—	—	✓	—	—	—	—
ROfFxAndAngle	—	—	—	✓	—	—	✓	—
ROfFxAndTime	—	—	—	✓	✓	—	✓	—
pMCROfRho	✓	—	—	—	—	—	—	—
pMCROfRhoAndTime	✓	—	—	—	✓	—	—	—
pMCROfXAndY	✓	—	✓	—	—	—	—	—
pMCROfXAndYAndTime	✓	—	✓	—	✓	—	—	—
pMCROfFx	—	—	—	✓	—	—	—	—
pMCROfFxAndTime	—	—	—	✓	✓	—	✓	—
dMCdROfRhoDMua	✓	—	—	—	—	—	—	—
dMCdROfRhoDMus	✓	—	—	—	—	—	—	—

**Table 3:** Partial list of MCCL history detector specifications identified by their name and options. The columns refer to the independent variables provided by these detectors in space (ρ,x,y,z), spatial-frequency ($f_{x}$), time (t), temporal-frequency (Ω), and/or polar and azimuthal angles (θ,ϕ).

History detector name	ρ∈[0,∞) [mm]	x,y∈(−∞,∞) [mm]	z∈[0,∞) [mm]	$f_{x}\in [0,\infty )$ $\left[{\mathrm{mm}}^{-1}\right]$	t∈[0,∞) [ns]	Ω∈[0,∞) [GHz]	θ∈[0,π] [rad]	ϕ∈[0,2π] [rad]
ATotal	—	—	—	—	—	—	—	—
AOfRhoAndZ	✓	—	✓	—	—	—	—	—
AOfXAndYAndZ	—	✓	✓	—	—	—	—	—
FluenceOfRhoAndZ	✓	—	✓	—	—	—	—	—
FluenceOfRhoAndZAndTime	✓	—	✓	—	✓	—	—	—
FluenceOfRhoAndZAndOmega	✓	—	✓	—	—	✓	—	—
FluenceOfXAndYAndZ	—	✓	✓	—	—	—	—	—
FluenceOfXAndYAndZAndTime	—	✓	✓	—	✓	—	—	—
FluenceOfXAndYAndZAndOmega	—	✓	✓	—	—	✓	—	—
RadianceOfRhoAndZAndAngle	✓	—	✓	—	—	—	✓	—
RadianceOfRhoAtZ	✓	—	✓	—	—	—	—	—
RadianceOfXAndYAndZAndThetaAndPhi	—	✓	✓	—	—	—	✓	✓
RadianceOfFxAndZAndAngle	—	—	✓	✓	—	—	✓	—
pMCATotal	—	—	—	—	—	—	—	—

### 3.4 Command Line Options

Once the downloadable MCCL zip file is extracted, typing command “mc help” (on Mac and Linux systems the command is “./mc help”) will display a list of command line options. Typing:

mc geninfiles

will generate example input files. Once input files have been generated, typing:

mc infile=infile_one_all_detectors.txt

will run MCCL with “infile_one_all_detectors.txt” which provides examples for how to define each of our detector types.

The command line environment provides the ability for the user to specify multiple MC simulations by defining a parameter sweep of desired inputs. For example, a user may wish to execute a given MC simulation for different combinations of optical properties with a fixed source, tissue, and detector configuration. The option “paramsweep=sweepParameter,start,stop,count” allows the user run simulations for varying $\mu_{a}$, $\mu_{s}$, g, and n values in any tissue layer. This is done by substituting the specified values into the input file and running the resulting simulations. The following example runs simulations using the “myinput” input file as the template with the $\mu_{a}$ value in layer 1 set to 0.01, 0.02, 0.03, 0.04, or 4 $\mu_{a}$ values within the range [0.01 to 0.04]. This single command launches four simulations with results found in folders that use the “OutputName” specified in the input file and appending the parameter sweep values. For example, setting “OutputName” to “test” in the input file

“OutputName”: “test,”

and using the command:

mc infile=myinput paramsweep=mua1,0.01,0.04,4

will generate output folders named “test_mua_0.01,” “test_mua_0.02,” “test_mua_0.03,” and “test_mua_0.04.” A sweep parameter “nphot” can designate the number of photons to launch

mc infile=myinput paramsweep=nphot,1000000,2000000,2

will execute two simulations, one with 1 million photons launched and the second with two million.

Also available are “paramsweepdelta” that uses a “delta” as the fourth parameter rather than a count, and “paramsweeplist” that can be used for values that are not equispaced. Equivalent commands to the paramsweep example using “paramsweepdelta” or “paramsweeplist” are:

mc infile=myinput paramsweepdelta=mua1,0.01,0.04,0.01

mc infile=myinput paramsweeplist=mua1,4,0.01,0.02,0.03,0.04

### 3.5 MCCL Outputs

**Fig. 3:**
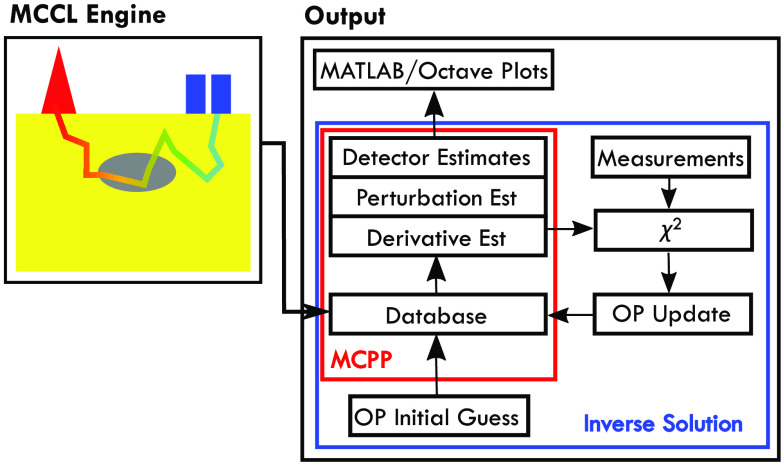
MC postprocessing and inverse solutions: (a) MCPP: has the ability to post-process a database to produce detector estimates and (b) inverse solution uses photon database and estimates of perturbation MC and differential MC within a gradient-based optimization routine to determine optical properties given a set of measurements.

The MCCL Engine produces binary files containing the detector tallies that can be plotted with MATLAB or Octave and/or binary database file(s) for further processing or analysis using our MCPP software (Fig. 3). The detector results are placed in a folder designated by the input file value for “OutputName.” Editing “load_results_script.m” and defining “datanames” to be the “OutputName” will define the file location with the results to be plotted. Executing the “load_results_script.m” at the MATLAB or Octave prompt will plot all detector results residing in the output folder. If only a subset of the detector results is desired, the list of toggles at the top of the script should be edited. For example, “show.ROfRho = 1” will provide visualization of only the results corresponding to that single detector. This script calls jsonlab code (https://github.com/fangq/jsonlab), an open-source MATLAB/Octave JSON encoder and decoder for Windows, Mac, and Linux, that is included with the zip file. If the user desires second moment data for any detector, they can set the “TallySecondMoment” flag to “true” for the corresponding detector. The second moment estimates can provide the standard deviation about the mean estimate for the detector. An example showing the screen display when MCCL is executed and the resulting plots, is provided on our MCCL GitHub wiki Sample Input and Output (https://github.com/VirtualPhotonics/Vts.MonteCarlo/wiki/MCCL-Sample-Input-And-Output).

The database output can be post-processed to produce any specified reflectance or transmittance detector result, perturbation MC reflectance estimates and/or derivatives of these estimates with respect to $\mu_{a}$ or $\mu_{s}$ to provide sensitivity results. The database can also be used within an inverse solution as shown in Fig. 3. The derivative estimates can be used within a nonlinear gradient optimization algorithm to resolve inverse problems. This algorithm executes a least-squared minimization between specified measurements and perturbation MC reflectance estimates to find tissue optical properties that would provide an MC prediction that best matches the measurement data.

Bundled with the downloadable MCCL zip file are MATLAB/Octave scripts to run inverse solutions on Linux, Windows, and Mac. Currently, MATLAB interop code to run inverse solutions is available on Windows only. We developed custom scripts that provide inverse solutions using pMC and dMC without using the interop code. Three examples of inverse solutions are provided in “mc_inverse.m”. The basic steps performed in the script are to: (1) create an input file that will generate a baseline database, (2) run that input file with MCCL, and (3) run an optimization algorithm using MATLAB “lsqcurvefit” or “fminsearch” for users that do not have access to the MATLAB Optimization Toolbox or are using Octave. The optimization algorithm requires a script that provides: (a) forward estimates for the updated parameters to be fit determined by pMC, and (b) the Jacobian of those estimates determined by dMC, both of which are generated by post-processing the database from the baseline simulation. A spectral database spreadsheet is included in the zip, “SpectralDictionary.xlsx”, that provides the spectra for oxyhemoglobin, deoxyhemoglobin,^30^ and water.^31^ The examples in “mc_inverse.m” include sample code to generate an inverse solution to (1) find $\mu_{a}$ and $\mu_{s}$ given spatially resolved measurements, (2) find absorber concentrations of ${\mathrm{HbO}}_{2}$, Hb, and water across 6 wavelengths, and (3) find absorber concentrations of Hb and ${\mathrm{HbO}}_{2}$ and power law $\mu_{s}^{\prime }=[A\lambda^{-b}]$ scatterer coefficients A and b given four wavelengths.

### 3.6 Validation and Comparison with MCML

Validation of our MC simulation engine is difficult because no exact RTE solutions exist for 3D problems. One approach to ensure that the MC simulations provide estimates that solve the RTE, is to derive the estimators from the integral form of the RTE. In previous publications, we have provided formulations for the reflectance estimators that we use in MCCL utilizing either discrete or continuous absorption weighting.^14^^,^^15^

While an exact RTE solution to a 3D problem is not available, an analytic solution is available for the 1D slab problem with bidirectional scattering.^32^ We have embedded unit tests within our software that use Analog and CAW random walk processes with bidirectional scattering to determine the total diffuse reflectance, total absorbance and total diffuse reflectance in a 1D refractive index matched slab. Our unit tests determine the MCCL mean and second moment estimates of these three detectors and verify (a) that the means are within 3σ of the respective analytic solutions, and (b) that the sum of these three detector tallies to 1. Table 4 displays the estimates for diffuse reflectance, total absorption, and diffuse transmittance using CAW as N increases, compared to the analytic solution. We specified optical properties $\mu_{a}=0.01/\mathrm{mm}$, $\mu_{s}^{\prime }=1/\mathrm{mm}$, g=0.8, n=1, and a 5-mm slab thickness. It is noteworthy that when N is increased by a factor of 100, the standard deviation reduces by a factor $\left(1/\sqrt{N}\right)$ providing another decimal digit of accuracy.

**Table 4:** Convergence to analytic solution as N, the number of photons launched, increases.

	Analytical	MCCL±1σ		
	Solution	$N=10^{2}$	$N=10^{4}$	$N=10^{6}$
Diffuse reflectance	0.68818	0.69809±0.04365	0.68569±0.00422	0.68760±0.00044
Absorption	0.04780	0.04930±0.00535	0.04771±0.00042	0.04781±0.00004
Diffuse transmittance	0.26402	0.25262±0.04063	0.26660±0.00419	0.26459±0.00042

We have also compared simulation results provided by MCCL with those provided by MCML^5^ for the case of a two-layer tissue illuminated by an collimated beam source. We compare estimates of specular reflection, total diffuse reflectance, total absorbance, total transmittance, and spatially resolved reflectance and absorbed energy in cylindrical coordinates. To perform this comparison, we used an MCML sample infile that defines a two-layered tissue with optical properties shown in Table 5. The number of photons launched by MCCL and MCML is $N=10^{6}$. MCML utilizes RR with a threshold of 0.0001. We ran the MCCL simulations with RR with the same threshold and without RR.

**Table 5:** Two-layer tissue optical properties used in comparison with MCML. Air is above tissue with n=1.0.

Layer	Thickness (mm)	$\mu_{a}$ (/mm)	$\mu_{s}$ (/mm)	g	n
1	0.1	2	20	0.7	1.3
2	∞	1	20	0.9	1.4

**Fig. 4:**
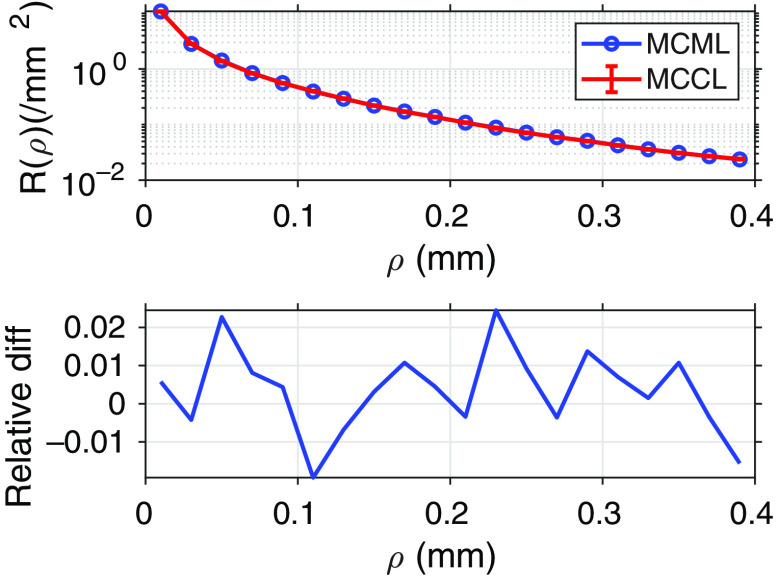
Comparison with MCML: plot of R(ρ) with RR turned on for both MCML and MCCL. One σ error bars for MCCL are smaller than symbols shown. The relative difference between the two results is plotted below.

Table 6 gives the results comparing MCML and MCCL with 1σ errors provided for MCCL. Figure 4 shows the plots of spatially resolved reflectance in cylindrical coordinates, R(ρ), with 1σ errors displayed for MCCL and the relative difference between the two plots. This comparison is documented on our MCCL GitHub wiki (https://github.com/VirtualPhotonics/Vts.MonteCarlo/wiki/MCCL-Validation-And-Comparison-With-MCML).

**Table 6:** Comparison of single valued tallies obtained using MCCL versus MCML.

Tally	MCML	MCCL with RR±1σ	MCCL without RR±1σ
Specular reflectance	0.0170	0.0171±0.0001	0.0168±0.0001
Diffuse reflectance	0.1050	0.1049±0.0002	0.1046±0.0002
Absorption	0.8780	0.8780±0.0002	0.8786±0.0002
Diffuse transmittance	0.0	0.0	0.0

## 4 Applications of MCCL

MCCL has been used to solve a variety of problems which include solving inverse problems using perturbation and differential MC, analyzing photon momentum transfer relevant to speckle imaging of flow, and determining the relative efficacy of port wine stain treatment modalities.

### 4.1 Inverse Solutions across Wavelength Using pMC/dMC

**Fig. 5:**
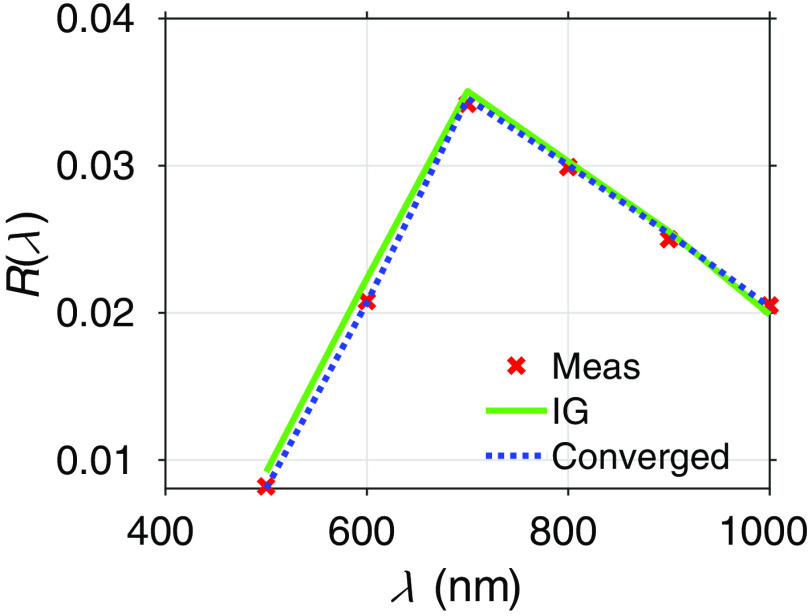
Inverse solution results produced by running mc_inverse.m using $N=10^{5}$ photons to determine chromophore concentrations across 6 wavelengths (Example 2 in “mc_inverse.m”).

The “mc_inverse.m” script described in Sec. 3.5 provides three examples of inverse solutions. The examples include solutions across wavelength to determine chromophore concentrations and/or scattering coefficients. Figure 5 shows the inverse solution results for Example 2 in “mc_inverse.m.” In this example, the inverse problem examined is the determination of concentrations of Hb, ${\mathrm{HbO}}_{2}$, and $H_{2}O$ given a set of measurements (Meas) at six wavelengths. The simulated measurements of reflectance as a function of wavelength were produced using a collimated source and scaled MC^33^ (red x’s). A database based on an initial guess (IG) of Hb (70  μM), ${\mathrm{HbO}}_{2}$ (30  μM), and $H_{2}O$ (80% volume fraction) was generated using $10^{5}$ photons. The algorithm generates derivatives of reflectance with respect to these chromophores to update this guess using a gradient-based optimization that minimizes the difference between the simulated measurements and perturbation MC estimates of the updated concentrations. After running the inverse solution the output at the conclusion of the inverse solution shows the actual concentrations of Hb, ${\mathrm{HbO}}_{2}$, and $H_{2}O$ that were used to generate the simulated measurements (Meas), the IG, and the value of $\chi^{2}$ at these values, the converged values of concentrations (Conv) and the associated $\chi^{2}$ value, and the error in the converged value compared to the actual.^34^

Meas = [72.000 35.000 0.600]

IG = [70.000 30.000 0.800] Chi2=4.946e-06

Conv = [75.468 35.707 0.620] Chi2=2.159e-07

error= [0.048 0.020 0.033]

These results show that the simulation of $10^{5}$ photons and six wavelengths is sufficient to provide an inverse solution using pMC and dMC with estimates of the actual chromophore concentrations with errors of <5%.

### 4.2 Momentum Transfer Analysis

**Fig. 6:**
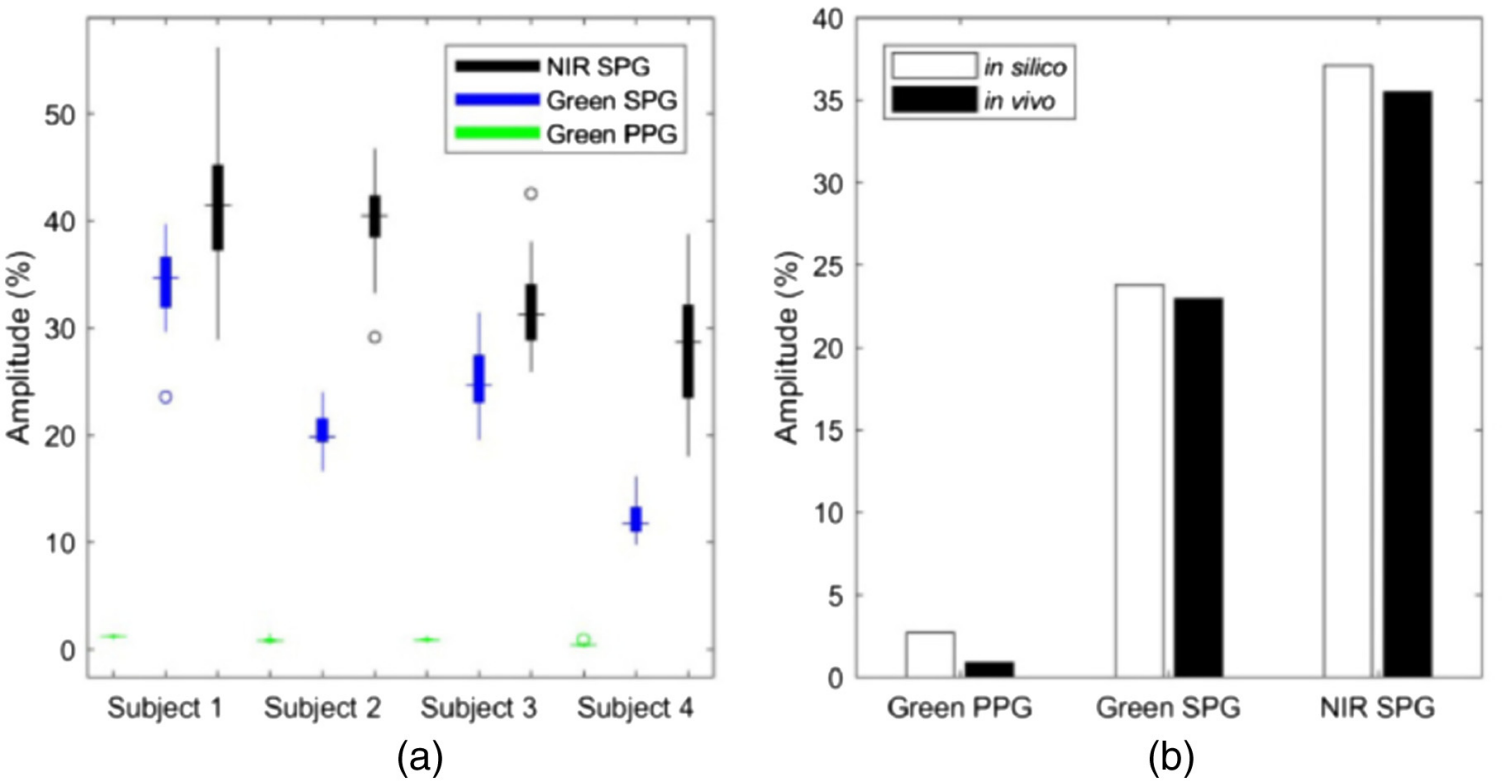
(a) NIR SPG, green SPG, and green PPG amplitude percentages from four subjects and (b) the MC estimates of the amplitude percent ranges (*in silico*) compared to the corresponding values across the subjects. Reprinted with permission from Ref. 35 © The Optical Society.

A multilayered tissue model was used to study the spectral and depth dependence of speckle contrast.^16^^,^^35^^,^^36^ Dunn et al.^35^ used MC momentum transfer detectors to compare speckleplethysmographic (SPG) with photoplethysmographic (PPG) imaging with *in vivo* measurements. The MC model consisted of a flat source, sufficiently broad to avoid edge effects, illuminating an eight-layer tissue model (epidermis, papillary dermis, upper blood net, reticular dermis, lower blood net, lipid, arterial layer, and lipid) using optical properties at wavelengths 532, 660, and 860 nm, and discrete absorption weighting. Dynamic momentum transfer detectors were utilized that allow the user to specify the blood volume fraction in each tissue region (ReflectedDynamicMTOfXAndYAndSubregionHistDetector). At each photon collision, the detector uses a uniformly distributed random number and compares it to the blood fraction of the region where the collision occurred to determine if the photon hit blood, and if so, tally to the momentum transfer for that region. MC simulations were executed on systems with the arterial layer expanded due to increased flow speed and contracted to simulate the pulsatility of arteries during systole and diastole, respectively, while compressing the upper and lower blood net layers accordingly to maintain a total thickness of 10 mm. The flow speed was varied within a range consistent with arterioles and capillaries. The MC detector estimates generate a probability distribution of momentum transfer that was used within a theoretical model to calculate speckle contrast using the correlation function. Figure 6(a) shows the SPG and PPG measurements taken from four subjects, and Fig. 6(b) comparison of the *in vivo* estimates with the *in silico* estimates provided by MC, which indicate good agreement.

### 4.3 Tissue Treatment Modality Comparison

The efficacy of intense pulsed light (IPL) to treat port wine stains using a broadband light spectrum approach compared to single wavelength (595 nm) pulsed dye laser (PDL) irradiation, was studied using MC estimates of absorbed energy in a vessel embedded in the hypodermis.^37^ Measurements were taken on an animal with a custom window chamber holder illuminated from the epidermal side with either the IPL or PDL. The MC model consisted of a flat circular source illuminating a four-layer system (skin, hypodermis, water and glass) with a vessel embedded in the hypodermis layer. An absorbed energy detector in Cartesian coordinates (AOfXAndYAndZDetector) and DAW were specified for the simulations which tallied deposited absorbed energy at each collision within a voxelized grid. For each simulation, $10^{6}$ photons were launched which ensured that the relative errors were <2%.

**Fig. 7:**
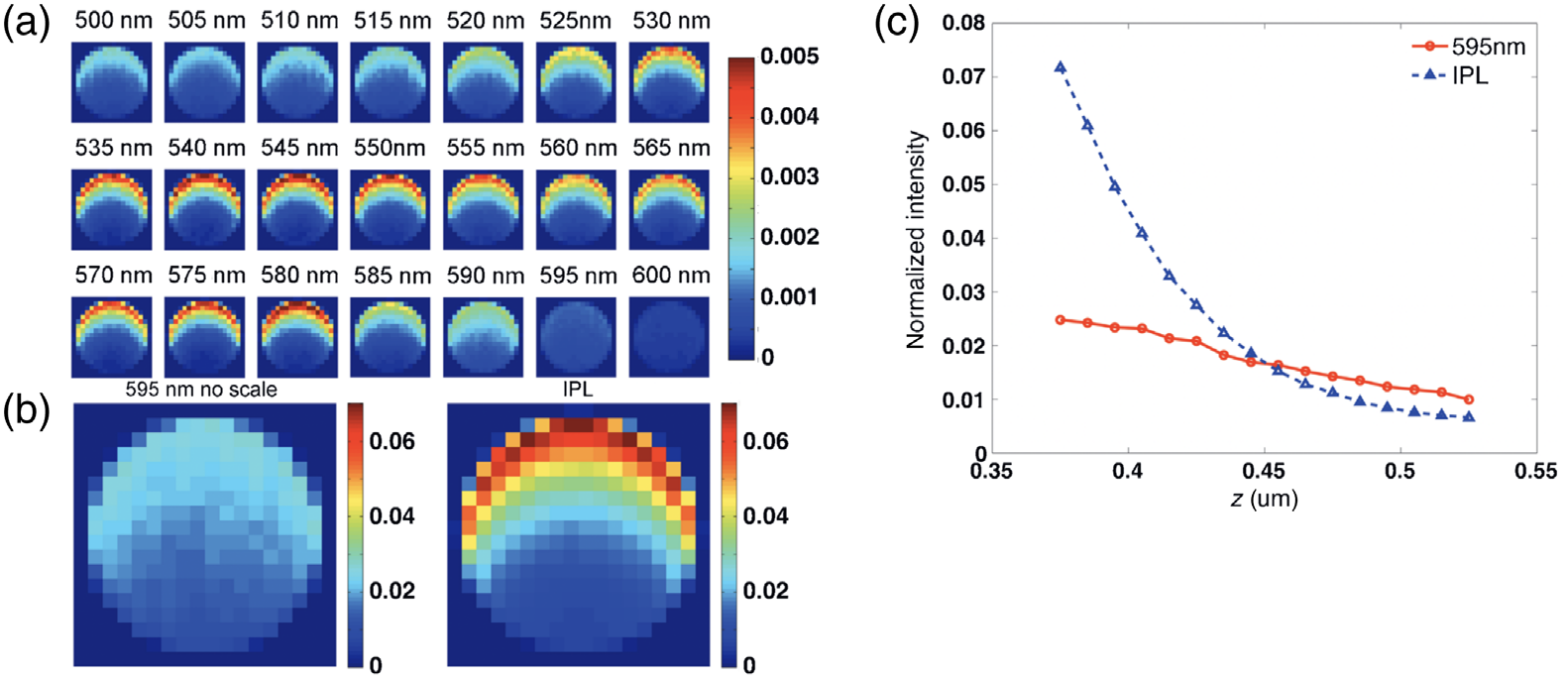
(a) Absorbed energy maps of a vessel over 500- to 600-nm range, (b) comparison of PDL and composite IPL, (c) line profile of absorbed energy distribution with 1σ error bars. Reprinted with permission from Ref. 37 © John Wiley and Sons.

Figure 7(a) shows the absorbed energy deposition for the wavelengths that comprise the IPL treatment, Fig. 7(b) the PDL versus IPL composite absorbed energy maps, and Fig. 7(c) a line profile along depth (z) with 1σ errorbars. These results indicate that the combined total absorbed energy induced by the IPL is higher compared to that of the PDL in the top half of the vessel. This result is consistent with experiments that showed the efficacy of IPL treatments to produce persistent vascular shutdown compared to PDL treatments.

## 5 Future Implementations

All software developed by the Virtual Photonics team is licensed under the MIT License and all repositories are on GitHub. Contributions are welcome. Any issues with our software can be described on the “Issues” tabs. We also have a Google Groups site (https://groups.google.com/g/virtual-photonics) where anyone can post questions or comments.
